# Wnt signaling in kidney: the initiator or terminator?

**DOI:** 10.1007/s00109-020-01978-9

**Published:** 2020-09-17

**Authors:** Ping Meng, Mingsheng Zhu, Xian Ling, Lili Zhou

**Affiliations:** 1 State Key Laboratory of Organ Failure Research, National Clinical Research Center of Kidney Disease, Division of Nephrology, Nanfang Hospital, Southern Medical University, 1838 North Guangzhou Ave, Guangzhou, 510515 China; 2 Department of Nephrology, Huadu District People’s Hospital, Southern Medical University, Guangzhou, China; 3 Department of Nephrology, The People’s Hospital of Gaozhou, Maoming, China; 4 Guangzhou Regenerative Medicine and Health Guangdong Laboratory, Guangzhou, China

**Keywords:** Wnt, Kidney, Development, Regeneration, Senescence

## Abstract

The kidney is a key organ in the human body that excretes toxins and sustains the water–electrolyte balance. During embryonic development and disease progression, the kidney undergoes enormous changes in macrostructure, accompanied by a variety of microstructural histological changes, such as glomerular formation and sclerosis, tubule elongation and atrophy, interstitial establishment, and fibrosis progression. All of these rely on the frequent occurrence of cell death and growth. Notably, to overcome disease, some cells regenerate through self-repair or progenitor cell differentiation. However, the signaling mechanisms underlying kidney development and regeneration have not been elucidated. Recently, Wnt signaling has been noted to play an important role. Although it is a well-known developmental signal, the role of Wnt signaling in kidney development and regeneration is not well recognized. In this review, we review the role of Wnt signaling in kidney embryonic development, tissue repair, cell division, and progenitor cell differentiation after injury. Moreover, we briefly highlight advances in our understanding of the pathogenic mechanisms of Wnt signaling in mediating cellular senescence in kidney parenchymal and stem cells, an irreversible arrest of cell proliferation blocking tissue repair and regeneration. We also highlight the therapeutic targets of Wnt signaling in kidney diseases and provide important clues for clinical strategies.

## Introduction

The kidney is a key organ in the human body that removes toxins and metabolic wastes by excreting urine. In addition, the kidney executes filtration and reabsorption, has endocrinal functions to regulate water–electrolyte balance, stabilize blood pressure, boost erythropoiesis, and promote bone formation and growth. Although weighing only about 0.5% of the total body weight, the kidney’s blood distribution occupies over 20% of the cardiac input [1]. To accomplish its tasks satisfactorily, the embryonic kidney develops extremely complex structures, including the nephron, peritubular capillary plexus, and interstitium [2]. Even though each kidney has a compensated brother, the kidney is still vulnerable to injury and age-related deterioration. Irrespective of the primary cause, various kidney diseases commonly involve the pathological feature of fibrosis [3, 4]. Notably, kidney fibrosis is an irreversible and intricate problem [5–7]. Furthermore, our knowledge of the underlying mechanisms still has vast gaps. Some developmental signals have caught the attention of scientists. Although silent after birth, those developmental signals may be utilized in the resurgence of boosting after disease. Among the numerous signals, Wnt signaling molecules undoubtedly stand at the forefront.

The Wnt family is a series of secretory glycoproteins with a cysteine-rich region. Through binding to frizzled (Fzd) receptor and the low-density lipid receptor family members Lrp5 and Lrp6 in the cell membrane, Wnt molecules transmit their signals. The Wnt signaling cascades are mainly triggered by the downstream effector β-catenin [8, 9]. In both embryonic and diseased stages, many Wnt family members participate in a multitude of cell regeneration and inactivation processes [10–12]. This evokes Wnt signaling as a double-edged sword initiating or terminating normal kidney function. In the present review, we focus on the role of Wnt signaling in kidney development and regeneration. Furthermore, we explore the role of Wnt signaling in cellular senescence, an irreversible arrest of cell proliferation blocking tissue repair and regeneration. We also highlight some therapeutic targets of Wnt signaling and provide clues for the development of novel clinical strategies.

## Wnt signaling in kidney development

Due to their complicated structure, kidneys undergo an important evolutionary process from embryonic intermediate mesoderm (IM) during development. In mammals, the embryonic kidney goes through three stages, i.e., the pronephros, the mesonephros, and the metanephros during maturation [13, 14]. There must be a series of signaling pathways involved in kidney development. Among the multiple signals, Wnt signaling, which is intimately involved in kidney development, is at a unique position.

As an evolutionary conserved signaling pathway, Wnt signaling plays an important role in low vertebrate pronephros and mammalian metanephros development [12]. Activated by the downstream canonical Wnt/β-catenin signaling pathway and the noncanonical Wnt signaling pathway, a number of Wnt molecules have been found to be intimately involved in nephrogenesis through the regulation of differentiation, proliferation, mesenchymal-to-epithelial trans-differentiation (MET), tubulogenesis, and morphogenesis [15–18]. However, its authentic role in kidney development is still not systematically clarified. Here, we review the role of Wnt signaling in kidney development, highlighting the important role of Wnt signaling in the metanephros in mammals.

### Kidney development

**Fig. 1:**
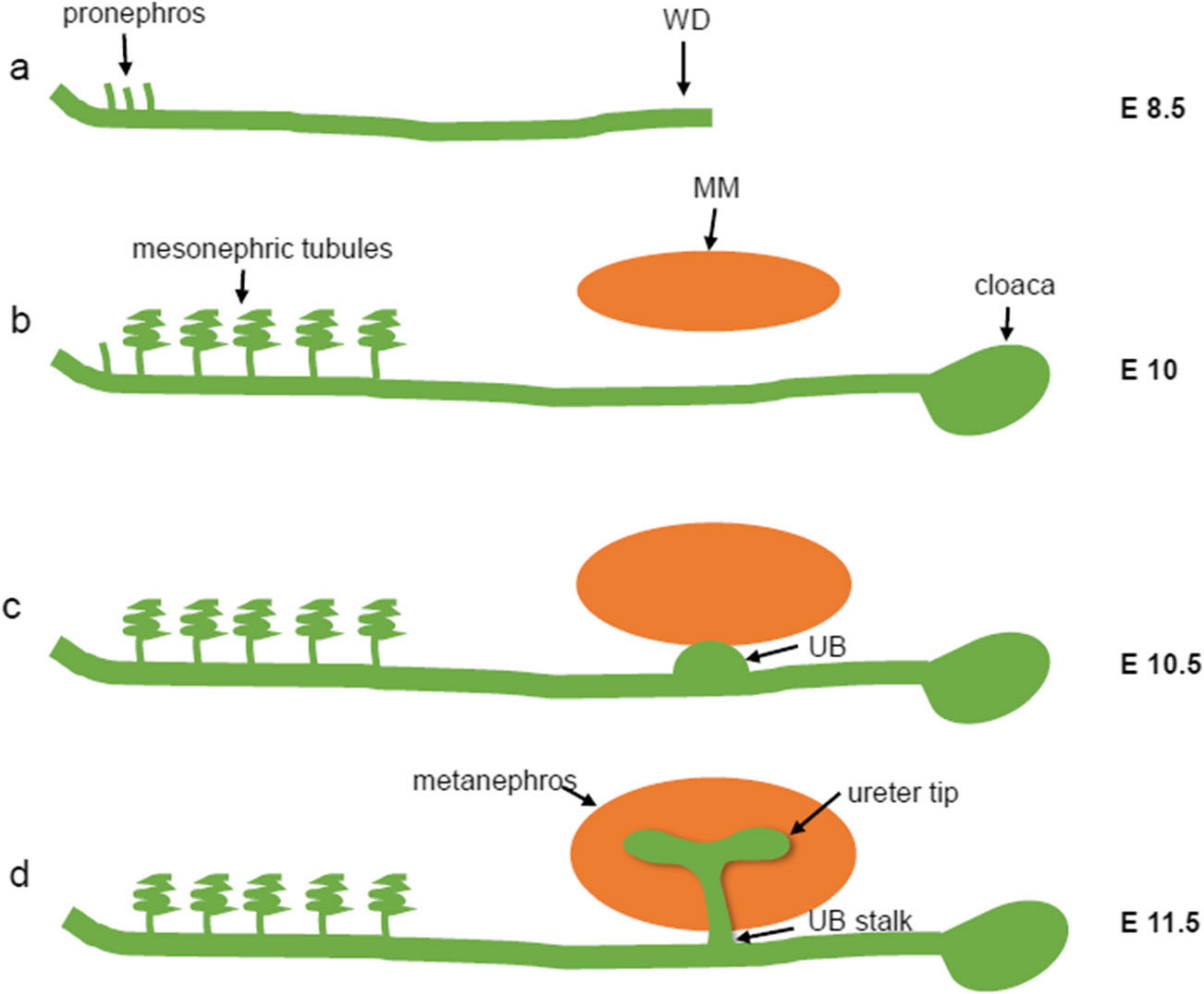
Schematic overview of kidney development in mice from E 8.5 to E11.5. **a** The pronephros originates from the IM at E 8.5 in mice. **b** The mesonephros tubules appear when the WD reaches the cloaca at E 10. **c** The UB forms from the WD and begins growing toward the MM. **d** As the UB branches into the MM, the metanephros is formed

**Fig. 2:**
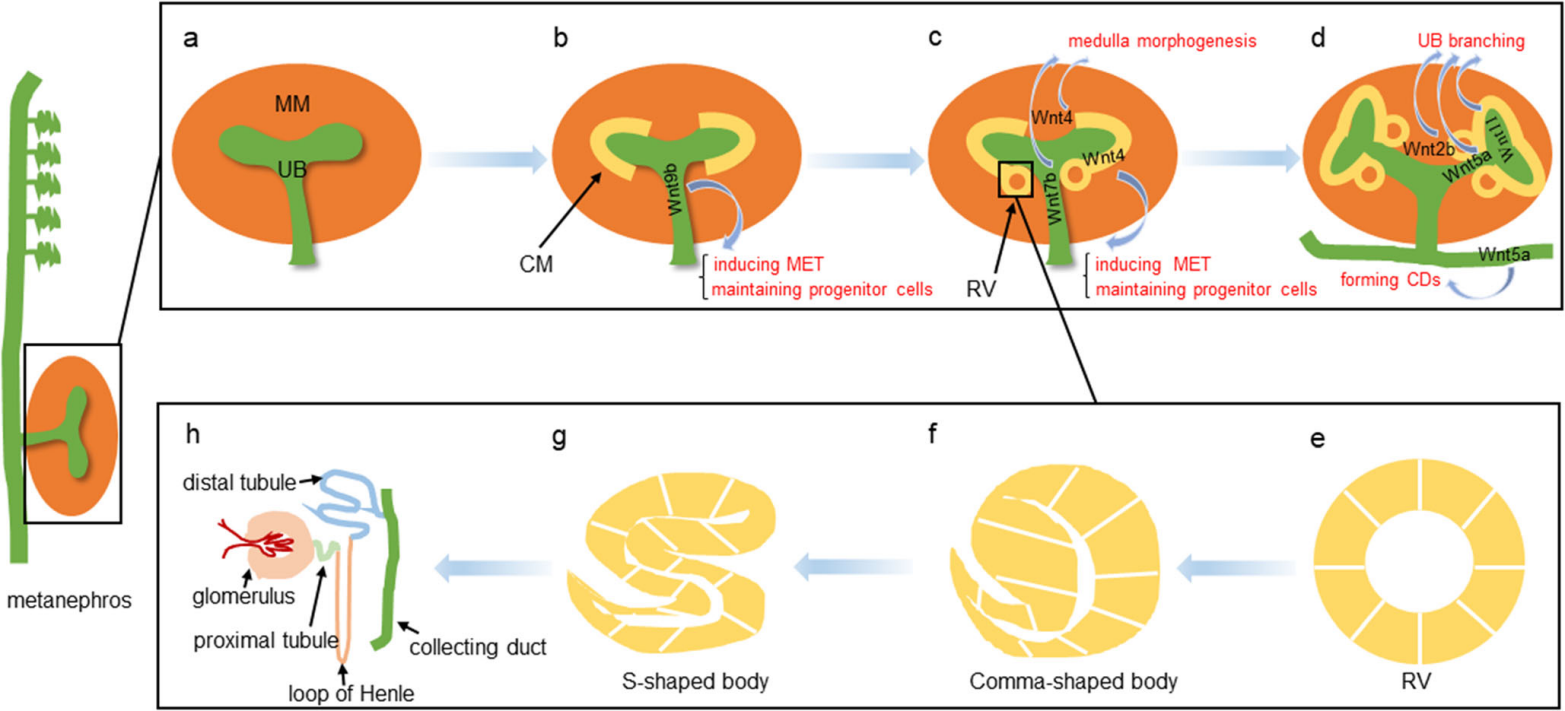
The locations and roles of Wnts in different nephron patterning during kidney development. **a** The metanephros is derived from the UB and MM. **b** CM is formed by the condensation of MM cells under the induction of UB. Wnt9b plays a crucial role in this process. **c** The RV is formed from the pretubular aggregate. Wnt4 and Wnt7b play important roles in medulla morphogenesis. In addition, Wnt4 is an inducer of MET and progenitor cell development. **d** UB branching morphogenesis. Wnt2b, Wnt5a, and Wnt11 are involved in this process. Besides, Wnt5a also has a role in the formation of CDs. **e–g** Nephron morphogenesis. Transition of the RV to a comma-shaped body and an S-shaped body. **h** The nephron consists of the glomerulus, the proximal tubules, the loop of Henle, the distal tubule, and the CDs

As a simple and transient structure, the pronephros develops with the emergence of paired nephric ducts (ND) at embryonic day 8.5 (E 8.5) in mice and about E 22 in humans [19–21]. At E 10 in mice, the mesonephric tubules appear, while at this time, the pronephric tubules are incompletely degraded, and the Wolffian duct (WD) reaches the cloaca [22]. The mesonephros develops from mesonephric tubules and has a transient function of filtration. One subset of the mesonephric tubules develops into the male reproductive system [23]. In human and murine organisms, the pronephros and mesonephros are transient organs and degenerate at the embryonic stage [2]. Unlike in lower vertebrates, such as zebrafish and Xenopus, where the pronephros is functional at the embryonic and larval stages [24, 25], the metanephros begins at E 32 in human and E 10.5 in mice [26]. Notably, the metanephros is permanent and will develop into a functional adult kidney in mammals. The metanephros is composed of ureteric bud (UB) and metanephric mesenchyme (MM). The UB protrudes from the caudal end of the mesonephric duct and invades MM to promote the development and maturation of the metanephros (Fig. 1). After formation of the cap mesenchyme (CM) by contact of UB cells with the MM, the UB develops into the collecting duct (CD) system under the induction of the MM. The MM also undergoes morphogenesis through MET [27], to form a comma-shaped body and further extend into an S-shaped body. The upper and middle branches of S-shaped corpuscles are distal tubules and proximal tubules. The lower branches form a scooped cyst-like structure, in which the lateral cells differentiate into glomerular parietal epithelial cells and the inner cells differentiate into podocytes. In addition, near the middle branches, the glomerular capillary loop forms after protruding of capillary globules into the ladle-like sac-like structure and eventually develops into a mature glomerulus (Fig. 2) [28–30]. Finally, the metanephros becomes the permanent and functional adult kidney in mammals.

### Role of Wnt signaling in pronephros development

Despite the transient expression in mammals, the pronephros in fish and amphibians is functional during their larval stages and consists of glomus, pronephric tubules, and pronephric duct [31, 32]. Several findings have implicated that Wnt signaling is involved in pronephros development. In zebrafish pronephros development, Wnt5a is located in the pronephric glomerulus and plays a role via non-canonical Wnt signaling pathway [33, 34]. Besides glomus, the development of nephric tubules is also tightly associated with Wnt signaling. A report showed that both Wnt11b and Wnt11 are mediators of pronephric tubule formation and play an important role in pronephros development in Xenopus [35]. In addition, Wnt4 is also critically required for pronephric tubulogenesis in Xenopus [36]. It has also been shown that DKK1, an inhibitor of Wnt/β-catenin signaling activation, leads to pronephric tubule formation failure in zebrafish. Other reports have also shown that Fzd receptors, including Fzd8 and Fzd3, take part in pronephric nephrogenesis. They act as vital players in the late differentiation of pronephric tubules and ducts [37] and trigger pronephric nephrogenesis via the non-canonical Wnt/JNK signaling pathway [38]. These findings reveal that Wnt signaling plays a key role in controlling pronephric development in lower vertebrate models.

### Role of Wnts in UB branching

Metanephros maturation relies on the mutual inductions between the UB and MM. First, the UB stimulates the MM to differentiate into the epithelia of glomeruli and renal tubules. In turn, the MM facilitates further branching of the UB and differentiation into CDs [39]. In UB branching, the ligands involved in Wnt signaling play central roles in their development.

Some studies suggest that Wnt11 is essentially involved in the UB branching. As early as E 8.25 in the mouse embryo, Wnt11 could be detected in the tips of the branching ureteric epithelium and is essential for metanephric kidney development especially early UB branching. Wnt11-deficient mice die within 2 days post-partum and have defects in UB branching, even if newborn mice have normal nephron organization and other phenotypes [40].

Besides Wnt11, Wnt5a is also necessary for metanephric kidney development. In mice, Wnt5a could be detected at the posterior end of the IM at E 9.5, in the whole area of the IM at E 10.5, in the WD/UB epithelia at E 11.0, and in the metanephros from E 13.5 to E 15.5. Wnt5a-deficient mice die shortly thereafter birth and exhibit duplicated ureters, medullary loss, renal agenesis, and horseshoe kidneys. In Wnt5a-deficient mice, the development of ureteric tree is affected by severely deregulated basement membrane organization. Furthermore, Wnt5a is crucial for the development of CDs derived from the kidney UB tree via bone marrow (BM) formation [41, 42]. Later, conditional Wnt5a-deficient mice have been generated; they exhibit the phenotype of bilateral duplex kidney and ureter formation in response to double UB outgrowth. In the mutant mice, the IM is also shortened and broadened. Thus, Wnt5a plays a role in IM development before the formation of the UB [43]. Both receptors of Wnt5a, Ror1 and Ror2, are primarily expressed in the mesenchyme between the MM and the WD, and are essential for UB outgrowth and branching morphogenesis during kidney development. In Ror1/Ror2 double mutant mice, the formation of UB is abnormal owing to the loss of MM. One report suggested that Wnt5a-Ror1/Ror2 is necessary for the formation of MM at the proper position in kidney organogenesis [44]. These results suggest that Wnt5a is important for promoting UB branching and CD formation.

Another Wnt protein, Wnt2b, previously called Wnt13, is widely expressed in the perinephric mesenchymal cells at E 11.5. As an early mesenchymal signal, Wnt2b directly stimulates UB branching during kidney development via regulating epithelial-mesenchymal transition (EMT). Notably, with the need for cooperation with kidney mesenchyme, Wnt2b induces UB branching morphogenesis and tubule formation [45]. These observations suggest that Wnt2b is a guiding signal to trigger mesenchyme activation. While Wnt2b is able to induce UB branching in in vitro experiments, there have been no reports about Wnt2b-deficient mice to directly show its roles in kidney development. Thus, the molecular mediators of Wnt2b in kidney development remain to be determined. Therefore, these genetic approaches have highlighted the critical roles of Wnt signaling in initiating UB branching and suggested deficiencies in several Wnts have been directly linked to serious kidney developmental defects.

### Role of Wnts in nephron maturation

Normal kidney function crucially relies on the proper development of nephrons. In mammals, the maturation of nephron mainly relies on nephron progenitors. After UB branching into the MM, the mesenchyme condenses and forms a cap-like structure, called CM, to cap the tips of the UB. CM cells are supposed to be in a signaling state and serve as the nephron progenitors [46]. Hence, by continuous branching of the UB and induction of the MM, the nephrons are driven to maturation with a fundamental composition of three parts: a glomerulus for blood filtration [47], a tubule that reabsorbs and secretes solutes, and a CD that transports the modified filtrate to a waste disposal site [48, 49]. Therefore, in mammals, new nephrons are formed from the mesenchymal progenitor cells, which are housed in the renal vesicle (RV) formed by the aggregation of the MM. Notably, Wnt signaling is the driving force for nephron progenitor cell (NPC) differentiation [50].

Several reports have shown that Wnt4 is necessary for kidney progenitor cell development. Wnt4 is first expressed in pretubular mesenchymal cells at E 11.5, and then in the epithelial CM, comma-shaped bodies, and S-shaped bodies. Of note, Wnt4 is essential for generating NPCs [51]. Reports have shown that Wnt4 is strongly expressed in NPCs, which originate from the pretubular cell aggregates. To induce mesenchyme differentiation and MET during nephrogenesis [51, 52], Wnt4 is crucial for the maturation of nephrons. Through modulating cell adhesion factors during kidney development [51], Wnt4 plays a critical role in tubule, mesenchymal, and ureteric epithelial structure formation [53–55].

Wnt9b, also known as Wnt14 or Wnt15, is abundant in the WD epithelium at E 9.5 and in the UB stalk from E 10.5 to E 14.5 [56]. Wnt9b-deficient mice lack intermediate progenitor cells and are embryonically lethal owing to the lack of mature nephrons and the large volume of peripheral mesenchyme and some rudimentary epithelia. As an autonomous activator to differentiate and renew progenitor cells, Wnt9b interacts with the adjacent RV and acts as the earliest inducer of MET in the organization of the urogenital system [57]. Wnt9b is also a key factor to regulate the balance of mesenchymal progenitor cell differentiation and cooperates with the signals from the stroma to promote differentiation of the progenitor cells [58, 59]. Besides its crucial role in the proliferation and commitment of progenitor cells, Wnt9b also has a direct action on tubule morphogenesis through a non-canonical Wnt/PCP signaling pathway [60].

Wnt11 is also involved in the normal nephrogenic program and contributes to the final kidney size. Wnt11 plays a direct role in the behavior of NPCs during kidney development [61]. Wnt11-mutant mice exhibit an accelerated and prematurely exhausted nephron progenitor pool, leading to disrupted tubular morphology, glomerular cysts, and smaller kidneys. Of note, Wnt11 is essential for the polarized distribution of NPCs. Wnt11 deficiency results in the abnormal dispersal of NPCs to intermix with interstitial progenitor cells, suggesting its important role in NPC programming. Faulty regulation of Wnt signaling results in defects and malformations in nephron maturation. Therefore, the unique and precise regulation of Wnt signaling is fundamental for NPCs.

### Role of Wnt receptors and β-catenin in kidney development

Though binding to the Fzd receptor and Lrp5 or Lrp6, Wnt signaling is started, leading to the activation of the downstream effector β-catenin. As important members of Wnt signaling, they are also required for nephric tubule morphogenesis [62, 63].

During kidney development, β-catenin is essential for mesenchymal progenitors to induce the formation of RV. The gain-of-function mutants of β-catenin are lethal within 24 h after birth, characterized by damaged epithelial branching and absence of tubulogenesis [64, 65].

Recently, studies also suggested that Fzd receptors such as Fzd7 and Fzd8 are expressed in the mesonephric duct and mesonephric tubules to play an important role in the morphogenetic movements of the proximal and intermediate tubules [66]. Interestingly, inversin, a molecular switch between Wnt signaling pathways also plays a role in regulating these processes. Besides Fzd receptors, Lrp6 is also intimately correlated with the embryonic kidney development. It has been reported that Lrp6-deficient mice present with abnormal kidneys with severe urogenital defects due to the inability of induction of UB branching [62]. These results suggest an indispensable role of Wnt/β-catenin signaling and its upstream receptors in kidney development.

### Wnt signaling in kidney medullary morphogenesis

The medulla of the adult kidney is responsible for regulating the concentration of urine [67] and is made up of medullary CDs, loops of Henle, vasa recta, and the interstitium. It is noteworthy that Wnt signaling is also involved in the development of these tissues.

Wnt7b is expressed in the stalk of the UB and unique for kidney medullary morphogenesis. Reports have shown that Wnt7b-deficient mice die at E 9.5 and fail to form the renal medulla, while kidney size and cortical development in Wnt7b homozygote mutants are not affected [68].

Furthermore, Wnt4 and several Wnt/β-catenin target genes are highly expressed in the medullary interstitium of the kidney [69]. Wnt4 is also expressed in the medullary stroma and plays an important role in kidney medullary morphogenesis. During embryonic kidney development, Wnt4 determines the fate of the smooth muscle cells in the medullary stroma and controls the differentiation of periureteric stroma cells [70].

In addition, nuclear β-catenin, an active form of β-catenin, is also strongly expressed in the medullary renal stromal cells. *β-cat*^*s−/−*^ mice, a conditional knockout mouse model with renal stroma-specific β-catenin deletion, exhibit impaired condensation of metanephros mesenchyme cells and reduced expression of Wnt9b in the ureter epithelium, which leads to abnormal MET [59]. Stromal mutation of β-catenin results in impaired nephrogenesis, blocking the differentiation of NPCs in the developing kidney [71].

## Wnt signaling in kidney regeneration

**Fig. 3:**
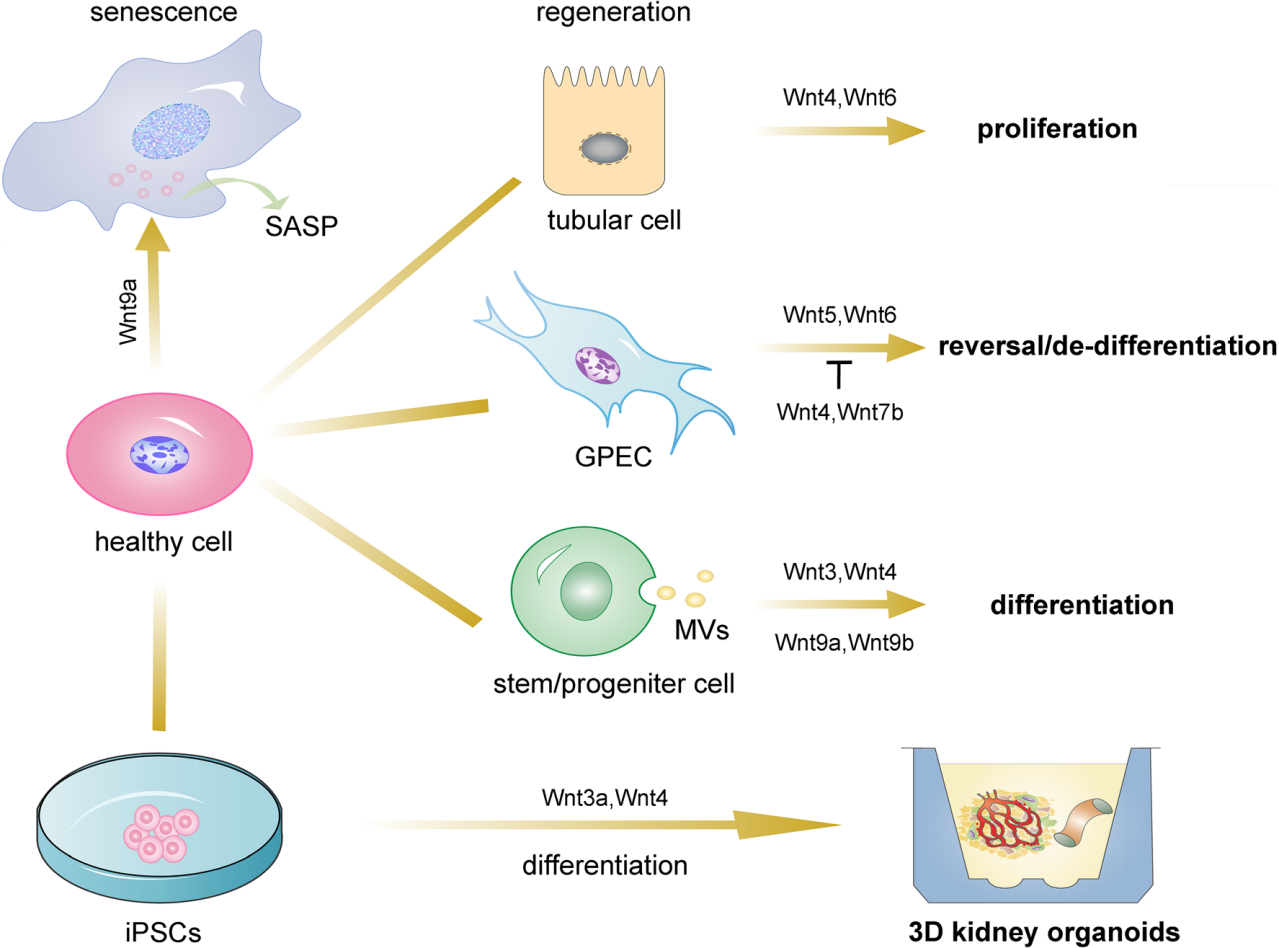
Role of Wnt signaling in kidney cellular senescence and regeneration. Wnt9a acts as an inducing factor in cellular senescence. On the contrary, Wnt signaling also plays an advantageous role in promoting regeneration in tubular cells, glomerular parietal cells, stem/progenitor cells, and 3D kidney organoids

The kidney is a key organ in the human body with complicated structures. In adult kidneys, it is difficult to regenerate the whole kidney for its diverse composition of different cells and sophisticated structures [72]. However, there is the hope that among the 25 distinct cell types, several kinds of cells still have limited abilities to self-repair or regenerate after injuries [73]. To date, there are three major strategies to achieve renal regeneration and repair: recruitment of circulating stem cells, differentiation of the somatic stem cells and resident cell de-differentiation or proliferation [74]. Notably, these processes are majorly involved in renal tubular cells, glomerular parietal cells, stem cells and renal progenitor cells, and 3D kidney organoids (Fig. 3).

### Renal tubular cells

Renal tubular cells are the major kidney parenchymal cells and are responsible for reabsorption and secretion. As epithelial cells, they are vulnerable to injury by strong stimuli and be induced to undergo apoptosis, necrosis, and senescence. However, renal tubular cells also possess the capability of self-repair or regeneration under mild stimulation. This is the reason why some acute kidney injury (AKI) patients could recover. Interestingly, Wnt signaling also plays a role in renal tubular cell recovery. Although only a few studies have been published, reports have shown that several Wnts are related to cell regeneration in renal tubular cells.

After AKI injury, renal Wnt4 expression is rapidly upregulated in the proximal tubules, inducing the upregulation of PCNA, cyclin D1 and cyclin A, which are cell proliferative markers [75]. This suggests that Wnt4 is responsible for tubular cell proliferation and repair. Consistently, modified pyrimidine,pyrimidine, a novel Wnt agonist, couldpromote cell proliferation by activating the Wnt/β-catenin/cyclin D1 pathway [76], and the a novel Wnt agonist, could promote cell proliferation by activating the Wnt/β-catenin/cyclin D1 pathway [76], and the specific ablation of β-catenin in renal tubular cells promotes apoptosis and aggravates kidney injury [77].

Another report has shown the effects of Wnt on renal tubular cell regeneration. Wnt6 is progressively lost after acute tubular injury. Ectopic expression of Wnt6 induces tubular cell epithelialization and triggers tubule formation in the 3D culture, through binding to the Fzd7 receptor [66, 78]. It follows that activation of Wnt/β-catenin is instrumental for tubular repair and regeneration after AKI.

### Glomerular parietal epithelial cells

Although located on the inner side of Bowman’s capsule, glomerular parietal epithelial cells (GPECs) are capable to migrate and differentiate into podocytes [79]. Furthermore, more and more studies have shown that GPECs have the characteristics of progenitor cells, as they simultaneously express CD133, and CD24, which are stem cell markers.

Interestingly, Wnt/β-catenin signaling is also necessary for parietal epithelial cell fate decision in embryo. During the late stages of nephrogenesis, β-catenin deficiency in renal epithelial cells at the late S-shaped body stage leads to the missing tubule layer, and especially, the failed lineage specification of GPECs. In the absence of Wnt/β-catenin, the proper differentiation of prospective GPECs is blocked and cells switch to the visceral, podocyte-specific cell fate. These direct lineages switch results in the small and underdeveloped capillary tufts. Finally, these conditional β-catenin knockout mice are born with abnormal kidneys and decreased kidney function [80].

Another interesting study shows that GPECs could regain the plasticity of kidney progenitors from terminally differentiated state, through EMT after renal injury. These reverted cells are expressed with both epithelial and mesenchymal markers, suggesting the de-differentiation of GPECs. Notably, these EMT-transformed GPECs express CD24, a kidney progenitor marker. Interestingly, some Wnts are potentially involved in this process. In the growth of these CD24+ cells, the levels of Wnt4 and Wnt7b, which are stimulators of renal epithelial integrity and proliferation, are significantly decreased; however, levels of Wnt5b and Wnt6 (expressed in the migratory mesenchymal cells) are highly increased. Consistently, the translocation of β-catenin from the cell membrane to the cytoplasm, a hallmark of EMT, also occurs in CD24+ cell propagation [81]. Although further studies are still needed, these results undoubtedly demonstrate the important role of Wnt/β-catenin signaling in the transition of GPECs to renal progenitors. Hence, more studies should be performed to clarify the subtle regulation of Wnt signaling on GPECs reverting into an embryonic phenotype. This would certainly provide new strategies to promote renal tissue repair and regeneration.

### Mesenchymal and progenitor cells

Stem cells are a class of cells with infinite self-renewal ability and could also differentiate into specialized somatic cells under the induction by the local microenvironment. There are several kinds of stem cells, such as mesenchymal stem cells (MSCs), pluripotent stem cells (PSCs), embryonic stem cells (ESCs), and induced PSCs (iPSCs). It is noteworthy that they have beneficial effects on kidney regeneration and therapeutic effects on kidney injury. Notably, Wnt signaling is an important self-renewal and differentiation factor for stem cells.

Reports have shown that bone marrow MSCs (BMSCs) complete the repair by releasing microvesicles containing an miRNA–mRNA network which can actively participate in the renal tubular epithelial cell regeneration via TGFβ–Wnt–EMT signaling [82, 83]. Furthermore, BMSC input could inhibit inflammation, which helps in the differentiation into renal tubular cells [84, 85]. Another report has shown that human PSCs could differentiate into mature podocytes through activation of the Wnt pathway [86]. Moreover, Wnt4-transfected ESCs differentiate into tubular structures expressing AQP2, a precursor form of tubule-like structures [87]. In addition, Wnt agonists such as CHIR99021, can stimulate differentiation in PSCs in the MM and UB to produce nephron structures which contain several different segments [88]. Notably, these iPSCs could generate renal organoids in vitro.

Progenitor cells are considered to be similar to stem cells, possessing differentiation potential, and are frequently called “endogenous stem cells”. Recently, renal progenitor cells have been recognized as the parietal cells distributed in Bowman’s capsule and renal tubular epithelium, especially the S3 fragment of the proximal tubule. The S3 segment of the proximal tubule is the most susceptible to acute tubule necrosis and could be found in urine. Reports have shown that Wnt signaling can promote urine-derived renal progenitor cells to differentiate into proximal tubule epithelium, indicating a new strategy for stimulating nephrogenesis [89]. In addition, Wnt3 exerts pro-regenerative effects and is upregulated in cisplatin-treated renal progenitor cells [90].

New nephrons are formed from Fzd9b and lef1 co-expressing progenitor cells in the zebrafish kidney, while the expression of Wnt ligands Wnt9a and Wnt9b is induced in distal tubules and CDs. Thus, the Wnt receptor Fzd9b is required to mediate Wnt signaling during zebrafish kidney regeneration [91].

### Three-dimensional kidney organoids

The technology of kidney organoids provides new avenues for recapitulating kidney developmental processes, modeling kidney disease pathogenesis, and holding great promise for patient-specific drug validation. Wnt signaling also plays an essential role in the process of three-dimensional (3D) kidney organoid formation [92].

A study has suggested that Wnt4 regulates nephrogenesis during kidney organoid development. There is no obvious visiblely morphological difference between wild-type and Wnt4^−/−^ organoids in the CRISPR/Cas9 knockout mouse ESC line except the failure to undergo nephrogenesis and formation of kidney structures [93]. Besides the role of epithelialization, Wnt4 also has a strong stimulatory effect on podocyte formation and differentiation from PSCs [94]. In addition, treatment with CHIR99021, a GSK3β inhibitor which activates Wnt signaling, can induce the generation of micro-organoids including patterning nephrons after 4 or 5 days in human PSCs. Meanwhile, qPCR confirmed that the expression of Wnt11 is upregulated [95]. Otherwise, stimulation with Wnt3a and EGF can result in the formation of branched tubular structures in intestinal epithelial cells [96].

These studies have shown several molecular, morphologic, and functional characteristics of 3D kidney organoids. In conclusion, Wnt signaling offers new opportunities for regenerative medicine and suggests that it may be critical to the regeneration of nephrons for the treatment of human kidney diseases. Although the importance of Wnt signaling has been reported, the molecular pathways still require more research.

## The role of Wnt signaling in kidney cellular senescence

Cellular senescence, induced by a number of stresses such as DNA damage, inflammation, and mitochondrial dysfunction, is a form of permanent cell cycle arrest. With a halted reactivation of proliferation, senescent cells undoubtedly bring a series of damage to kidneys. It is noteworthy that in the early stages of clinical nephropathies, senescent cells accumulate. These senescent cells not only lose their capacity to regenerate and replicate DNA, but also release cytokines of the senescence-associated secretory phenotype (SASP) to negatively influence neighboring cells. More and more reports have shown that kidney diseases involve a clinical manifestation of accelerated senescence. However, the underlying signaling mechanisms remain to be elucidated. Recently, our group and others reported that Wnt signaling reactivation is intimately associated with cellular senescence in adult kidneys [97–99].

Our recent studies showed that Wnt9a is upregulated in various chronic kidney diseases and responsible for renal tubular cell senescence (Fig. 3). These senescent tubular cells show growth arrest and have lost their epithelial characteristics. Besides that, they can also secrete SASP cytokines to accelerate renal fibrosis [100, 101]. Hence, the modulation of senescent cells is critical for controlling the outcome of kidney diseases. In fact, cellular senescence is also thought to contribute to tissue aging in vivo. Interestingly, our most recent studies showed that Klotho, an endogenous inhibitor of Wnt signaling, exhibits beneficial effects in age-related kidney fibrosis and the associated mitochondrial dysfunction [102, 103]. However, the detailed role of Wnt signaling in kidney cellular senescence still requires further study.

Although cellular senescence has been implicated as a major cause of aging-related diseases, it can also play a positive role. It is noteworthy that cellular senescence is also associated with mammalian kidney development and vertebrate pronephros development. Senescent cells are activated by Wnt signaling pathways during mesonephros development [104–106]. However, whether Wnt signaling is essential in kidney developmental senescence is still not clear.

## Therapeutic potentials of targeting Wnt signaling

**Fig. 4:**
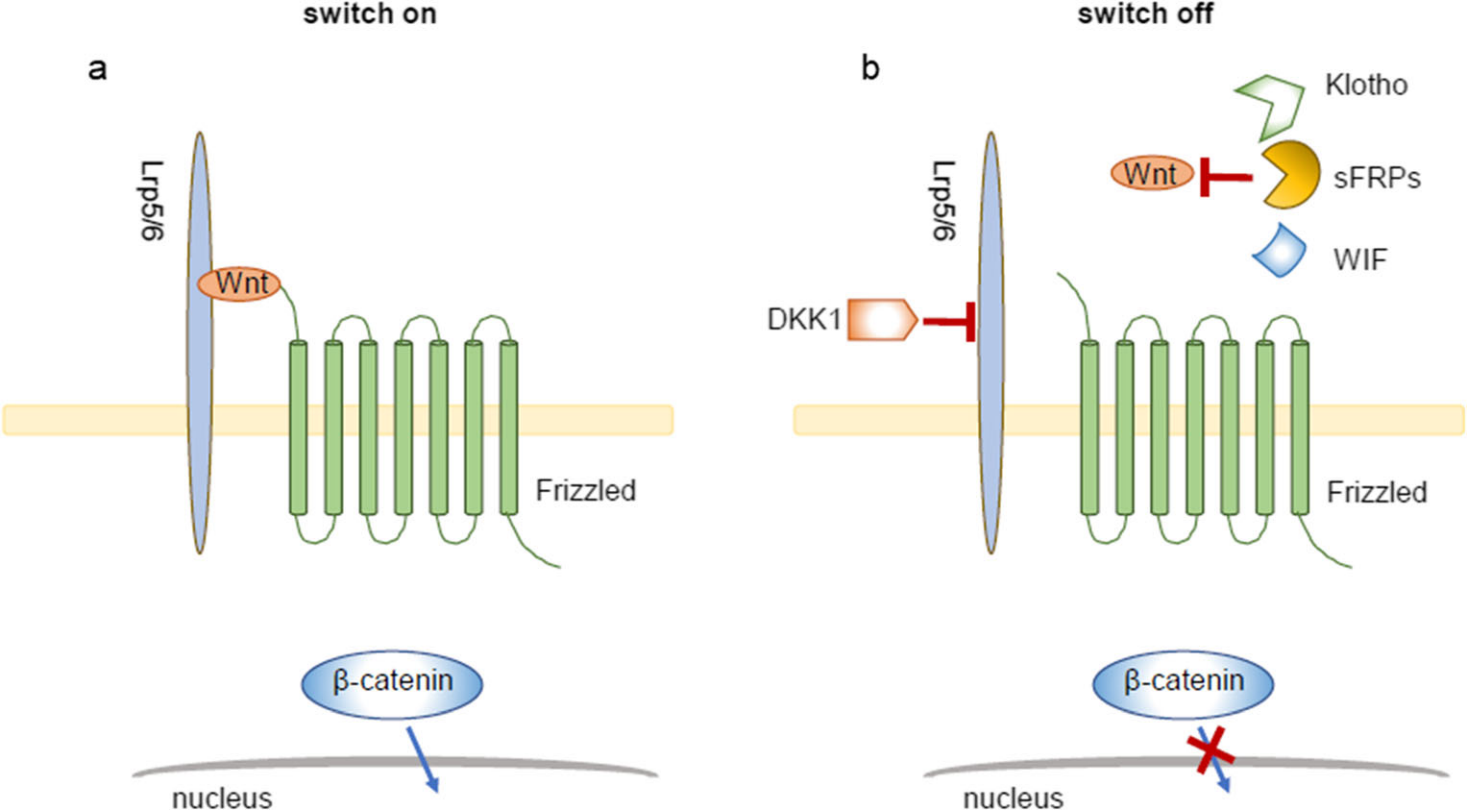
Therapeutic targets against canonical Wnt signaling. Wnt signaling is regulated at the receptor-ligand level. Klotho, sFRPs, and WIF inhibit Wnt signaling through direct binding to Wnt ligands. DKK1 binds to Lrp5/6, and then disrupts Wnt signaling. Wnt signaling is switched on upon binding to Fzd receptor and its co-receptor Lrp5/6, which leads to the translocation of β-catenin and transcription of downstream genes. Due to the presence of these Wnt antagonists (Klotho, sFRPs, WIF, and DKK1), Wnt signaling is switched off, which leads to the retention of β-catenin in the cytoplasm

In the embryonic or acute injury stage, Wnt signaling plays an important role in the normal programming of development and cell regeneration. However, the reactivation of Wnt signaling in adult kidneys is majorly responsible for the deterioration of chronic kidney diseases (CKDs) [101, 107–111], suggesting its double-edged role in kidney diseases. The sustained activation of Wnt signaling in kidneys would promote disease progression [112–115], through the induction of cellular senescence, clarifying the inhibitory pathway by which retarding Wnt signaling is of significance to the treatment of CKDs. Although the inhibitory factors of Wnt signaling still need more investigations, there are some promising inhibitors of Wnt signaling at the receptor-ligand level such as Klotho, Dickkopf1 (DKK1), secreted Fzd-related proteins (sFRPs), and Wnt inhibitory factor (WIF) (Fig. 4). Targeting Wnt signaling with these inhibitors holds promise for future treatment of CKD patients.

### Klotho

Klotho has been identified decades ago as an abundantly expressed anti-aging protein in normal adult kidneys. Klotho deficiency is highly related to the progression of CKD [116, 117]. Interestingly, our recent studies have verified that Klotho is also an endogenous Wnt antagonist. The secreted form of Klotho possesses the capability of binding to multiple Wnt ligands such as Wnt1, Wnt4, and Wnt7a, to further block the activation of β-catenin, the downstream effector of Wnt signaling. It could be speculated that Klotho blocks the binding of Wnt ligand to its receptor Lrp. Hence, as an inhibitor of Wnt signaling at the ligand level, Klotho has potent inhibitory effects on Wnt signaling. Therefore, it is not surprising that increased expression of Klotho can reduce renal fibrosis and improve the kidney function [115, 118].

Excitingly, Klotho is also beneficial to stem cell renewal and survival. Klotho-modified MSCs have superior therapeutic effects in AKI that are associated with the inhibition of Wnt/β-catenin signaling [119]. Further studies have also shown that adipose-derived MSC transplantation promotes Klotho expression in diabetic nephropathy, blocks Wnt/β-catenin signaling, and attenuates renal injury [120]. These results suggest that Klotho and stem cells mutually reinforce each other. Hence, as stem cells possess the ability of self-renewal and differentiation, exogenous supplementation of Klotho could provide greater therapeutic potentials in kidney diseases beyond its inhibitory function on Wnt signaling. However, the role of Klotho in kidney stem/progenitor cells still needs further study.

### DKK1

The *DKK* gene family encodes secretory proteins including DKK1 to DKK4 [121]. DKK1 is a major member of the family and acts as a secretory Wnt signaling antagonist through disrupting Wnt binding to its co-receptors Lrp5/6 [122, 123]. In kidney development, DKK1 inhibits UB branching and nephron formation via canonical Wnt signaling, and together with Wnt7b, it controls the morphogenesis of renal medulla [124]. Another study has suggested that Wnt7b is important for kidney repair and regeneration. DKK1 significantly suppresses the activities of Wnt7b produced by macrophage from the injured kidney, while DKK2 enhances the repair process [125]. However, mounting evidence indicates that DKK1 could protect against renal fibrosis and inflammation via inhibiting Wnt signaling [126], suggesting its potential role in retarding chronic kidney diseases.

### Secreted Fzd-related proteins

Secreted Fzd-related proteins (sFRPs), a heterogeneous family of five secreted glycoproteins (sFRP1 to sFRP5) in mammals, are antagonists of Wnt signaling. Several studies have suggested that sFRPs directly bind to Wnt ligands to block their interaction with receptors. Indeed, the expression of each sFRP varies in different way. Through acting on non-canonical Wnt/PCP signaling, sFRP1 protects against kidney damage and inhibit the increase in myofibroblast counts [127]. Furthermore, sFRP3 and sFRP5 act as tumor suppressor genes, delaying the progression of carcinogenesis in kidneys [128]. Interestingly, recent studies have shown that sFRP5 inhibits high phosphate-induced calcification in vascular smooth muscle cells, an important indicator of CKD-mineral and bone disorder [129, 130]. All of these pieces of evidences indicate their potential application value in kidney diseases. However, the high application value of sFRPs in kidney diseases needs to be further explored.

### Wnt inhibitory factor

Wnt inhibitory factor (WIF-1) is another secretory protein that has been proposed as one of the Wnt antagonists. WIF-1 directly binds to Wnts and blocks the binding of Wnts to receptors [131]. Most studies involve tumors and suggest that WIF-1 is a tumor suppressor. WIF-1 overexpression could inhibit kidney tumor cell growth and promote apoptosis through inhibiting Wnt signaling activity [132]. However, the role of WIF-1 in other CKDs is still unclear and should be fully investigated. Manipulation of this signaling pathway by diverse strategies may eventually translate into effective treatments for patients with various kidney disorders.

## Conclusions

In summary, we have presented a general review of our current knowledge of the role of Wnt signaling in kidney embryonic development and regeneration after injury. Wnt signaling plays a critical role in nephric tubules development and NPC maturation and guidance, and is a critical factor in normal kidney organogenesis. However, the reactivation of Wnt signaling is highly related to the progression of kidney diseases and cellular senescence. Of note, some Wnts are still beneficial for the induction of tubular cell repair, GPEC differentiation, and mesenchyme stem cell self-renewal. These observations suggest that Wnt signaling serves as a double-edged sword from the initial to the final stage in kidney development and disease progression.

Considering there are 19 Wnt members in mammals, this heterogeneity of Wnt effects must be related to the effects of every single Wnt member. In addition to its myriad of roles in kidney development and regeneration, Wnt signaling also takes part in tubular cellular senescence and the aging process, highlighting that it is necessary to maintain balanced Wnt signaling activity in the kidney. Wnt signaling plays a complex role in exerting both positive and negative effects in the kidney. Although much remains to be discovered in this research area, there is the hope that the iceberg under the sea surface will gradually be revealed. Nevertheless, future studies should focus on the therapeutic target potential of Wnt signaling, optimally modulating the balance in Wnt signaling, and taking into account both benefits and disadvantages, in kidneys. This will bring us one step closer to the development of effective treatment strategies for kidney diseases in the human population.

## References

[1] Ebefors K, Nystrom J (2017). New insights into crosstalk in the kidney. Curr Opin Nephrol Hypertens.

[2] Lindstrom NO, McMahon JA, Guo J, Tran T, Guo Q, Rutledge E, Parvez RK, Saribekyan G, Schuler RE, Liao C, Kim AD, Abdelhalim A, Ruffins SW, Thornton ME, Baskin L, Grubbs B, Kesselman C, McMahon AP (2018). Conserved and divergent features of human and mouse kidney organogenesis. J Am Soc Nephrol.

[3] Zhou L, Zhou S, Yang P, Tian Y, Feng Z, Xie XQ, Liu Y (2018). Targeted inhibition of the type 2 cannabinoid receptor is a novel approach to reduce renal fibrosis. Kidney Int.

[4] Mo H, Wu Q, Miao J, Luo C, Hong X, Wang Y, Tang L, Hou FF, Liu Y, Zhou L (2017). C-X-C chemokine receptor type 4 plays a crucial role in mediating oxidative stress-induced podocyte injury. Antioxid Redox Signal.

[5] Zuo Y, Liu Y(2018) New insights into the role and mechanism of Wnt/beta-catenin signalling in kidney fibrosis. Nephrology (Carlton) 23 Suppl 4**:**38-43 10.1111/nep.13472 30298654

[6] Tan RJ, Zhou D, Zhou L, Liu Y(2014) Wnt/beta-catenin signaling and kidney fibrosis**.** Kidney Int Suppl (2011) 4**:**84–90 10.1038/kisup.2014.16 PMC4536962 26312156

[7] Li Z, Zhou L, Wang Y, Miao J, Hong X, Hou FF, Liu Y (2017). (Pro)renin receptor is an amplifier of Wnt/beta-catenin signaling in kidney injury and fibrosis. J Am Soc Nephrol.

[8] Niehrs C (2012). The complex world of WNT receptor signalling. Nat Rev Mol Cell Biol.

[9] Ramakrishnan AB, Cadigan KM (2017). Wnt target genes and where to find them. F1000Res.

[10] Zhou D, Tan RJ, Fu H, Liu Y (2016). Wnt/beta-catenin signaling in kidney injury and repair: a double-edged sword. Lab Investig.

[11] Zhou L, Liu Y (2015). Wnt/beta-catenin signalling and podocyte dysfunction in proteinuric kidney disease. Nat Rev Nephrol.

[12] Wang Y, Zhou CJ, Liu Y (2018). Wnt signaling in kidney development and disease. Prog Mol Biol Transl Sci.

[13] Brown AC, Muthukrishnan SD, Guay JA, Adams DC, Schafer DA, Fetting JL, Oxburgh L (2013). Role for compartmentalization in nephron progenitor differentiation. Proc Natl Acad Sci U S A.

[14] Challen G, Gardiner B, Caruana G, Kostoulias X, Martinez G, Crowe M, Taylor DF, Bertram J, Little M, Grimmond SM (2005). Temporal and spatial transcriptional programs in murine kidney development. Physiol Genomics.

[15] Nusse R, Varmus H (2012). Three decades of Wnts: a personal perspective on how a scientific field developed. EMBO J.

[16] Merkel CE, Karner CM, Carroll TJ (2007). Molecular regulation of kidney development: is the answer blowing in the Wnt?. Pediatr Nephrol.

[17] Halt K, Vainio S (2014). Coordination of kidney organogenesis by Wnt signaling. Pediatr Nephrol.

[18] McCoy KE, Zhou X, Vize PD (2011). Non-canonical wnt signals antagonize and canonical wnt signals promote cell proliferation in early kidney development. Dev Dyn.

[19] Walker KA, Bertram JF (2011). Kidney development: core curriculum 2011. Am J Kidney Dis.

[20] Pietilä I, Vainio SJ (2014). Kidney Development: An Overview. Nephron Exp Nephrol.

[21] de Bakker BS, van den Hoff M, Vize PD, Oostra RJ (2019). The pronephros; a fresh perspective. Integr Comp Biol.

[22] McMahon AP(2016) Development of the mammalian kidney 10.1016/bs.ctdb.2015.10.010 PMC5007134 26969971

[23] Reidy KJ, Rosenblum ND (2009). Cell and molecular biology of kidney development. Semin Nephrol.

[24] Outtandy P, Russell C, Kleta R, Bockenhauer D (2019). Zebrafish as a model for kidney function and disease. Pediatr Nephrol.

[25] Krneta-Stankic V, DeLay BD, Miller RK (2017). Xenopus: leaping forward in kidney organogenesis. Pediatr Nephrol.

[26] Dressler GR (2009). Advances in early kidney specification, development and patterning. Development.

[27] Schmidt-Ott KM, Lan D, Hirsh BJ, Barasch J (2006). Dissecting stages of mesenchymal-to-epithelial conversion during kidney development. Nephron Physiology.

[28] Yu J, McMahon AP, Valerius MT (2004). Recent genetic studies of mouse kidney development. Curr Opin Genet Dev.

[29] Saxen L, Sariola H (1987). Early organogenesis of the kidney. Pediatr Nephrol.

[30] Miner JH (2014). Organogenesis of the kidney glomerulus. Organogenesis.

[31] Wessely O, Tran U (2011). Xenopus pronephros development--past, present, and future. Pediatr Nephrol.

[32] Naylor RW, Qubisi SS, Davidson AJ (2017). Zebrafish pronephros development. Results Probl Cell Differ.

[33] Huang L, Xiao A, Choi SY, Kan Q, Zhou W, Chacon-Heszele MF, Ryu YK, McKenna S, Zuo X, Kuruvilla R, Lipschutz JH (2014). Wnt5a is necessary for normal kidney development in Zebrafish and mice. Nephron Exp Nephrol.

[34] Huang L, Xiao A, Wecker A, McBride DA, Choi SY, Zhou W, Lipschutz JH (2014) A possible zebrafish model of polycystic kidney disease: knockdown of wnt5a causes cysts in zebrafish kidneys**.** J Vis Exp 10.3791/52156 PMC4354438 25489842

[35] Tételin S, Jones EA (2009). Xenopus Wnt11b is identified as a potential pronephric inducer.

[36] Saulnier DM, Ghanbari H, Brandli AW (2002). Essential function of Wnt-4 for tubulogenesis in the Xenopus pronephric kidney. Dev Biol.

[37] Satow R, Chan TC, Asashima M (2004). The role of Xenopus frizzled-8 in pronephric development. Biochem Biophys Res Commun.

[38] Cizelsky W, Tata A, Kuhl M, Kuhl SJ (2014). The Wnt/JNK signaling target gene alcam is required for embryonic kidney development. Development.

[39] Glass NR, Takasako M, Er PX, Titmarsh DM, Hidalgo A, Wolvetang EJ, Little MH, Cooper-White JJ (2020). Multivariate patterning of human pluripotent cells under perfusion reveals critical roles of induced paracrine factors in kidney organoid development. Sci Adv.

[40] Majumdar A (2003). Wnt11 and Ret/Gdnf pathways cooperate in regulating ureteric branching during metanephric kidney development. Development.

[41] Yamaguchi TP, Bradley A, McMahon AP, Jones S (1999). A Wnt5a pathway underlies outgrowth of multiple structures in the vertebrate embryo. Development.

[42] Pietilä I, Prunskaite-Hyyryläinen R, Kaisto S, Tika E, van Eerde AM, Salo AM, Garma L, Miinalainen I, Feitz WF, Bongers EMHF, Juffer A, Knoers NVAM, Renkema KY, Myllyharju J, Vainio SJ (2016). Wnt5a deficiency leads to anomalies in ureteric tree development, tubular epithelial cell organization and basement membrane integrity pointing to a role in kidney collecting duct patterning. PLoS One.

[43] Yun K, Ajima R, Sharma N, Costantini F, Mackem S, Lewandoski M, Yamaguchi TP, Perantoni AO (2014). Non-canonical Wnt5a/Ror2 signaling regulates kidney morphogenesis by controlling intermediate mesoderm extension. Hum Mol Genet.

[44] Qi X, Okinaka Y, Nishita M, Minami Y (2016). Essential role of Wnt5a-Ror1/Ror2 signaling in metanephric mesenchyme and ureteric bud formation. Genes Cells.

[45] Lin Y, Liu A, Zhang S, Ruusunen T, Kreidberg JA, Peltoketo H, Drummond I, Vainio S (2001). Induction of ureter branching as a response to Wnt-2b signaling during early kidney organogenesis. Dev Dyn.

[46] Costantini F, Kopan R (2010). Patterning a complex organ: branching morphogenesis and nephron segmentation in kidney development. Dev Cell.

[47] Takasato M, Er PX, Becroft M, Vanslambrouck JM, Stanley EG, Elefanty AG, Little MH (2014). Directing human embryonic stem cell differentiation towards a renal lineage generates a self-organizing kidney. Nat Cell Biol.

[48] Bertram JF, Douglas-Denton RN, Diouf B, Hughson MD, Hoy WE (2011). Human nephron number: implications for health and disease. Pediatr Nephrol.

[49] Short KM, Combes AN, Lefevre J, Ju AL, Georgas KM, Lamberton T, Cairncross O, Rumballe BA, McMahon AP, Hamilton NA, Smyth IM, Little MH (2014). Global quantification of tissue dynamics in the developing mouse kidney. Dev Cell.

[50] Park JS, Ma W, O'Brien LL, Chung E, Guo JJ, Cheng JG, Valerius MT, McMahon JA, Wong WH, McMahon AP (2012). Six2 and Wnt regulate self-renewal and commitment of nephron progenitors through shared gene regulatory networks. Dev Cell.

[51] Stark K, Vainio S, Vassileva G, McMahon AP (1994). Epithelial transformation of metanephric mesenchyme in the developing kidney regulated by Wnt-4. Nature.

[52] Shan J, Jokela T, Skovorodkin I, Vainio S (2010). Mapping of the fate of cell lineages generated from cells that express the Wnt4 gene by time-lapse during kidney development☆. Differentiation.

[53] Tanigawa S, Wang H, Yang Y, Sharma N, Tarasova N, Ajima R, Yamaguchi TP, Rodriguez LG, Perantoni AO (2011). Wnt4 induces nephronic tubules in metanephric mesenchyme by a non-canonical mechanism. Dev Biol.

[54] Georgas K, Rumballe B, Valerius MT, Chiu HS, Thiagarajan RD, Lesieur E, Aronow BJ, Brunskill EW, Combes AN, Tang D, Taylor D, Grimmond SM, Potter SS, McMahon AP, Little MH (2009). Analysis of early nephron patterning reveals a role for distal RV proliferation in fusion to the ureteric tip via a cap mesenchyme-derived connecting segment. Dev Biol.

[55] Kispert A, Vainio S, McMahon AP (1998). Wnt-4 is a mesenchymal signal for epithelial transformation of metanephric mesenchyme in the developing kidney. Development.

[56] Qian J, Jiang Z, Li M, Heaphy P, Liu Y, Shackleford GM (2003). Mouse Wnt9b transforming activity, tissue-specific expression, and evolution. Genomics.

[57] Carroll TJ, Park J, Hayashi S, Majumdar A, McMahon AP (2005). Wnt9b plays a central role in the regulation of mesenchymal to epithelial transitions underlying organogenesis of the mammalian urogenital system. Dev Cell.

[58] Karner CM, Das A, Ma Z, Self M, Chen C, Lum L, Oliver G, Carroll TJ (2011). Canonical Wnt9b signaling balances progenitor cell expansion and differentiation during kidney development. Development.

[59] Boivin FJ, Sarin S, Lim J, Javidan A, Svajger B, Khalili H, Bridgewater D (2015). Stromally expressed beta-catenin modulates Wnt9b signaling in the ureteric epithelium. PLoS One.

[60] Karner CM, Chirumamilla R, Aoki S, Igarashi P, Wallingford JB, Carroll TJ (2009). Wnt9b signaling regulates planar cell polarity and kidney tubule morphogenesis. Nat Genet.

[61] O'Brien LL, Combes AN, Short KM, Lindström NO, Whitney PH, Cullen-McEwen LA, Ju A, Abdelhalim A, Michos O, Bertram JF, Smyth IM, Little MH, McMahon AP (2018) Wnt11 directs nephron progenitor polarity and motile behavior ultimately determining nephron endowment. Elife 7 10.7554/eLife.40392 PMC6281319 30516471

[62] Wang Y, Stokes A, Duan Z, Hui J, Xu Y, Chen Y, Chen HW, Lam K, Zhou CJ (2016). LDL receptor-related protein 6 modulates Ret proto-oncogene signaling in renal development and cystic dysplasia. J Am Soc Nephrol.

[63] Ye X, Wang Y, Rattner A, Nathans J (2011). Genetic mosaic analysis reveals a major role for frizzled 4 and frizzled 8 in controlling ureteric growth in the developing kidney. Development.

[64] Park JS, Valerius MT, McMahon AP (2007). Wnt/beta-catenin signaling regulates nephron induction during mouse kidney development. Development.

[65] Sarin S, Boivin F, Li A, Lim J, Svajger B, Rosenblum ND, Bridgewater D (2014). beta-Catenin overexpression in the metanephric mesenchyme leads to renal dysplasia genesis via cell-autonomous and non-cell-autonomous mechanisms. Am J Pathol.

[66] Beaton H, Andrews D, Parsons M, Murphy M, Gaffney A, Kavanagh D, McKay GJ, Maxwell AP, Taylor CT, Cummins EP, Godson C, Higgins DF, Murphy P, Crean J (2016). Wnt6 regulates epithelial cell differentiation and is dysregulated in renal fibrosis. Am J Physiol Ren Physiol.

[67] Song R, Yosypiv IV (2012). Development of the kidney medulla. Organogenesis.

[68] Yu J, Carroll TJ, Rajagopal J, Kobayashi A, Ren Q, McMahon AP (2008). A Wnt7b-dependent pathway regulates the orientation of epithelial cell division and establishes the cortico-medullary axis of the mammalian kidney. Development.

[69] Pile T, Raftery M, Thuraisingham R, Kirwan CJ, Harwood S, Yaqoob MM (2020). Treating posttransplant anemia with erythropoietin improves quality of life but does not affect progression of chronic kidney disease. Exp Clin Transplant.

[70] Itaranta P, Chi L, Seppanen T, Niku M, Tuukkanen J, Peltoketo H, Vainio S (2006). Wnt-4 signaling is involved in the control of smooth muscle cell fate via Bmp-4 in the medullary stroma of the developing kidney. Dev Biol.

[71] Drake KA, Chaney CP, Das A, Roy P, Kwartler CS, Rakheja D, Carroll TJ (2020). Stromal beta-catenin activation impacts nephron progenitor differentiation in the developing kidney and may contribute to Wilms tumor. Development.

[72] Elger M, Hentschel H, Litteral J, Wellner M, Kirsch T, Luft FC, Haller H (2003). Nephrogenesis is induced by partial nephrectomy in the elasmobranch Leucoraja erinacea. J Am Soc Nephrol.

[73] Little MH, Combes AN (2019). Kidney organoids: accurate models or fortunate accidents. Genes Dev.

[74] Anglani F, Ceol M, Mezzabotta F, Torregrossa R, Tiralongo E, Tosetto E, Del PD, D'Angelo A (2008). The renal stem cell system in kidney repair and regeneration. Front Biosci.

[75] Terada Y, Tanaka H, Okado T, Shimamura H, Inoshita S, Kuwahara M, Sasaki S (2003). Expression and function of the developmental gene Wnt-4 during experimental acute renal failure in rats. J Am Soc Nephrol.

[76] He YX, Diao TT, Song SM, Wang CC, Wang Y, Zhou CL, Bai YB, Yu SS, Mi X, Yang XY, Wei QJ, Li B (2018). Wnt4 is significantly upregulated during the early phases of cisplatin-induced acute kidney injury. Sci Rep.

[77] Zhou D, Li Y, Lin L, Zhou L, Igarashi P, Liu Y (2012). Tubule-specific ablation of endogenous beta-catenin aggravates acute kidney injury in mice. Kidney Int.

[78] Denby L, Conway BR (2016). Wnt6: another player in the yin and yang of renal Wnt signaling. Am J Physiol Ren Physiol.

[79] Appel D, Kershaw DB, Smeets B, Yuan G, Fuss A, Frye B, Elger M, Kriz W, Floege J, Moeller MJ (2009). Recruitment of podocytes from glomerular parietal epithelial cells. J Am Soc Nephrol.

[80] Grouls S, Iglesias DM, Wentzensen N, Moeller MJ, Bouchard M, Kemler R, Goodyer P, Niggli F, Grone HJ, Kriz W, Koesters R (2012). Lineage specification of parietal epithelial cells requires beta-catenin/Wnt signaling. J Am Soc Nephrol.

[81] Swetha G, Chandra V, Phadnis S, Bhonde R (2011). Glomerular parietal epithelial cells of adult murine kidney undergo EMT to generate cells with traits of renal progenitors. J Cell Mol Med.

[82] Bruno S, Grange C, Deregibus MC, Calogero RA, Saviozzi S, Collino F, Morando L, Busca A, Falda M, Bussolati B, Tetta C, Camussi G (2009). Mesenchymal stem cell-derived microvesicles protect against acute tubular injury. J Am Soc Nephrol.

[83] de Almeida DC, Bassi EJ, Azevedo H, Anderson L, Origassa CS, Cenedeze MA, de Andrade-Oliveira V, Felizardo RJ, Da SR, Hiyane MI, Semedo P, Dos RM, Moreira-Filho CA, Verjovski-Almeida S, Pacheco-Silva A, Camara NO (2016). A regulatory miRNA-mRNA network is associated with tissue repair induced by mesenchymal stromal cells in acute kidney injury. Front Immunol.

[84] Bi B, Schmitt R, Israilova M, Nishio H, Cantley LG (2007). Stromal cells protect against acute tubular injury via an endocrine effect. J Am Soc Nephrol.

[85] Togel F, Weiss K, Yang Y, Hu Z, Zhang P, Westenfelder C (2007). Vasculotropic, paracrine actions of infused mesenchymal stem cells are important to the recovery from acute kidney injury. Am J Physiol Ren Physiol.

[86] Qian T, Hernday SE, Bao X, Olson WR, Panzer SE, Shusta EV, Palecek SP (2019). Directed differentiation of human pluripotent stem cells to podocytes under defined conditions. Sci Rep.

[87] Kobayashi T, Tanaka H, Kuwana H, Inoshita S, Teraoka H, Sasaki S, Terada Y (2005). Wnt4-transformed mouse embryonic stem cells differentiate into renal tubular cells. Biochem Biophys Res Commun.

[88] Takasato M, Er PX, Chiu HS, Maier B, Baillie GJ, Ferguson C, Parton RG, Wolvetang EJ, Roost MS, Lopes SM, Little MH (2016). Kidney organoids from human iPS cells contain multiple lineages and model human nephrogenesis. Nature.

[89] Rahman MS, Wruck W, Spitzhorn LS, Nguyen L, Bohndorf M, Martins S, Asar F, Ncube A, Erichsen L, Graffmann N, Adjaye J (2020). The FGF, TGFbeta and WNT axis modulate self-renewal of human SIX2(+) urine derived renal progenitor cells. Sci Rep.

[90] Brossa A, Papadimitriou E, Collino F, Incarnato D, Oliviero S, Camussi G, Bussolati B (2018). Role of CD133 molecule in Wnt response and renal repair. Stem Cells Transl Med.

[91] Kamei CN, Gallegos TF, Liu Y, Hukriede N, Drummond IA (2019). Wnt signaling mediates new nephron formation during zebrafish kidney regeneration. Development.

[92] Low JH, Li P, Chew E, Zhou B, Suzuki K, Zhang T, Lian MM, Liu M, Aizawa E, Rodriguez EC, Yong K, Chen Q, Campistol JM, Fang M, Khor CC, Foo JN, Izpisua BJ, Xia Y (2019). Generation of human PSC-derived kidney organoids with patterned nephron segments and a de novo vascular network. Cell Stem Cell.

[93] Tan Z, Shan J, Rak-Raszewska A, Vainio SJ (2018). Embryonic stem cells derived kidney organoids as faithful models to target programmed nephrogenesis. Sci Rep.

[94] Yoshimura Y, Taguchi A, Tanigawa S, Yatsuda J, Kamba T, Takahashi S, Kurihara H, Mukoyama M, Nishinakamura R (2019). Manipulation of nephron-patterning signals enables selective induction of podocytes from human pluripotent stem cells. J Am Soc Nephrol.

[95] Kumar SV, Er PX, Lawlor KT, Motazedian A, Scurr M, Ghobrial I, Combes AN, Zappia L, Oshlack A, Stanley EG, Little MH (2019) Kidney micro-organoids in suspension culture as a scalable source of human pluripotent stem cell-derived kidney cells. Development:146 10.1242/dev.172361 PMC6432662 30846463

[96] Matsumoto S, Fujii S, Sato A, Ibuka S, Kagawa Y, Ishii M, Kikuchi A (2014). A combination of Wnt and growth factor signaling induces Arl4c expression to form epithelial tubular structures. EMBO J.

[97] Song YY, Miao JH, Qin FY, Yan YM, Yang J, Qin DP, Hou FF, Zhou LL, Cheng YX (2018). Belamchinanes A-D from Belamcanda chinensis: triterpenoids with an unprecedented carbon skeleton and their activity against age-related renal fibrosis. Org Lett.

[98] Regulski MJ (2017). Cellular senescence: what, why, and how. Wounds.

[99] Liu H, Fergusson MM, Castilho RM, Liu J, Cao L, Chen J, Malide D, Rovira II, Schimel D, Kuo CJ, Gutkind JS, Hwang PM, Finkel T (2007). Augmented Wnt signaling in a mammalian model of accelerated aging. Science.

[100] Xiong Y, Zhou L (2019). The signaling of cellular senescence in diabetic nephropathy. Oxidative Med Cell Longev.

[101] Luo C, Zhou S, Zhou Z, Liu Y, Yang L, Liu J, Zhang Y, Li H, Liu Y, Hou FF, Zhou L (2018). Wnt9a promotes renal fibrosis by accelerating cellular senescence in tubular epithelial cells. J Am Soc Nephrol.

[102] Stenvinkel P, Larsson TE (2013). Chronic kidney disease: a clinical model of premature aging. Am J Kidney Dis.

[103] Miao J, Liu J, Niu J, Zhang Y, Shen W, Luo C, Liu Y, Li C, Li H, Yang P, Liu Y, Hou FF, Zhou L (2019). Wnt/beta-catenin/RAS signaling mediates age-related renal fibrosis and is associated with mitochondrial dysfunction. Aging Cell.

[104] Munoz-Espin D, Canamero M, Maraver A, Gomez-Lopez G, Contreras J, Murillo-Cuesta S, Rodriguez-Baeza A, Varela-Nieto I, Ruberte J, Collado M, Serrano M (2013). Programmed cell senescence during mammalian embryonic development. Cell.

[105] Davaapil H, Brockes JP, Yun MH (2017). Conserved and novel functions of programmed cellular senescence during vertebrate development. Development.

[106] Storer M, Mas A, Robert-Moreno A, Pecoraro M, Ortells MC, Di Giacomo V, Yosef R, Pilpel N, Krizhanovsky V, Sharpe J, Keyes WM (2013). Senescence is a developmental mechanism that contributes to embryonic growth and patterning. Cell.

[107] Kim S, Nie H, Nesin V, Tran U, Outeda P, Bai CX, Keeling J, Maskey D, Watnick T, Wessely O, Tsiokas L (2016). The polycystin complex mediates Wnt/Ca(2+) signalling. Nat Cell Biol.

[108] Zhou D, Fu H, Zhang L, Zhang K, Min Y, Xiao L, Lin L, Bastacky SI, Liu Y (2017). Tubule-derived Wnts are required for fibroblast activation and kidney fibrosis. J Am Soc Nephrol.

[109] Chen S, Fu H, Wu S, Zhu W, Liao J, Hong X, Miao J, Luo C, Wang Y, Hou FF, Zhou L, Liu Y (2019). Tenascin-C protects against acute kidney injury by recruiting Wnt ligands. Kidney Int.

[110] Gewin LS (2018) Renal tubule repair: is Wnt/beta-catenin a friend or foe? Genes (Basel) 9 10.3390/genes9020058 PMC5852554 29364168

[111] Zhou L, Li Y, He W, Zhou D, Tan RJ, Nie J, Hou FF, Liu Y (2015). Mutual antagonism of Wilms' tumor 1 and beta-catenin dictates podocyte health and disease. J Am Soc Nephrol.

[112] Xiao L, Zhou D, Tan RJ, Fu H, Zhou L, Hou FF, Liu Y (2016). Sustained activation of Wnt/beta-catenin signaling drives AKI to CKD progression. J Am Soc Nephrol.

[113] Zhou L, Chen X, Lu M, Wu Q, Yuan Q, Hu C, Miao J, Zhang Y, Li H, Hou FF, Nie J, Liu Y (2019). Wnt/beta-catenin links oxidative stress to podocyte injury and proteinuria. Kidney Int.

[114] Zhou L, Li Y, Hao S, Zhou D, Tan RJ, Nie J, Hou FF, Kahn M, Liu Y (2015). Multiple genes of the renin-angiotensin system are novel targets of Wnt/beta-catenin signaling. J Am Soc Nephrol.

[115] Zhou L, Mo H, Miao J, Zhou D, Tan RJ, Hou FF, Liu Y (2015). Klotho ameliorates kidney injury and fibrosis and normalizes blood pressure by targeting the renin-angiotensin system. Am J Pathol.

[116] Zou D, Wu W, He Y, Ma S, Gao J (2018). The role of klotho in chronic kidney disease. BMC Nephrol.

[117] Hu MC, Shi M, Zhang J, Quinones H, Griffith C, Kuro-o M, Moe OW (2011). Klotho deficiency causes vascular calcification in chronic kidney disease. J Am Soc Nephrol.

[118] Zhou L, Li Y, Zhou D, Tan RJ, Liu Y (2013). Loss of Klotho contributes to kidney injury by derepression of Wnt/beta-catenin signaling. J Am Soc Nephrol.

[119] Zhang F, Wan X, Cao YZ, Sun D, Cao CC (2018). Klotho gene-modified BMSCs showed elevated antifibrotic effects by inhibiting the Wnt/beta-catenin pathway in kidneys after acute injury. Cell Biol Int.

[120] Ni W, Fang Y, Xie L, Liu X, Shan W, Zeng R, Liu J, Liu X (2015). Adipose-derived mesenchymal stem cells transplantation alleviates renal injury in streptozotocin-induced diabetic nephropathy. J Histochem Cytochem.

[121] Niehrs C (2006). Function and biological roles of the Dickkopf family of Wnt modulators. Oncogene.

[122] He X, Semenov M, Tamai K, Zeng X (2004). LDL receptor-related proteins 5 and 6 in Wnt/beta-catenin signaling: arrows point the way. Development.

[123] Baetta R, Banfi C (2019). Dkk (Dickkopf) Proteins. Arterioscler Thromb Vasc Biol.

[124] Pietila I, Ellwanger K, Railo A, Jokela T, Barrantes IB, Shan J, Niehrs C, Vainio SJ (2011). Secreted Wnt antagonist Dickkopf-1 controls kidney papilla development coordinated by Wnt-7b signalling. Dev Biol.

[125] Lin SL, Li B, Rao S, Yeo EJ, Hudson TE, Nowlin BT, Pei H, Chen L, Zheng JJ, Carroll TJ, Pollard JW, McMahon AP, Lang RA, Duffield JS (2010). Macrophage Wnt7b is critical for kidney repair and regeneration. Proc Natl Acad Sci U S A.

[126] Johnson BG, Ren S, Karaca G, Gomez IG, Fligny C, Smith B, Ergun A, Locke G, Gao B, Hayes S, MacDonnell S, Duffield JS (2017). Connective tissue growth factor domain 4 amplifies fibrotic kidney disease through activation of LDL receptor-related protein 6. J Am Soc Nephrol.

[127] Warr N, Siggers P, Bogani D, Brixey R, Pastorelli L, Yates L, Dean CH, Wells S, Satoh W, Shimono A, Greenfield A (2009). Sfrp1 and Sfrp2 are required for normal male sexual development in mice. Dev Biol.

[128] Nikuseva-Martic T, Serman L, Zeljko M, Vidas Z, Gasparov S, Zeljko HM, Kosovic M, Pecina-Slaus N (2013). Expression of secreted frizzled-related protein 1 and 3, T-cell factor 1 and lymphoid enhancer factor 1 in clear cell renal cell carcinoma. Pathol Oncol Res.

[129] Kawakami K, Yamamura S, Hirata H, Ueno K, Saini S, Majid S, Tanaka Y, Kawamoto K, Enokida H, Nakagawa M, Dahiya R (2011). Secreted frizzled-related protein-5 is epigenetically downregulated and functions as a tumor suppressor in kidney cancer. Int J Cancer.

[130] Deng D, Diao Z, Han X, Liu W (2016). Secreted frizzled-related protein 5 attenuates high phosphate-induced calcification in vascular smooth muscle cells by inhibiting the Wnt/ss-catenin pathway. Calcif Tissue Int.

[131] Kerekes K, Banyai L, Trexler M, Patthy L (2019). Structure, function and disease relevance of Wnt inhibitory factor 1, a secreted protein controlling the Wnt and hedgehog pathways. Growth Factors.

[132] Kawakami K, Hirata H, Yamamura S, Kikuno N, Saini S, Majid S, Tanaka Y, Kawamoto K, Enokida H, Nakagawa M, Dahiya R (2009). Functional significance of Wnt inhibitory factor-1 gene in kidney cancer. Cancer Res.

